# Long-term Health Risks of Cancer in Ukraine: Insights from the 2011 Fukushima Triple Disaster Experience

**DOI:** 10.31662/jmaj.2022-0154

**Published:** 2022-11-30

**Authors:** Yudai Kaneda, Akihiko Ozaki, Hiroaki Saito, Toyoaki Sawano, Masaharu Tsubokura

**Affiliations:** 1 School of Medicine, Hokkaido University, Sapporo, Japan; 2 Department of Breast and Thyroid Surgery, Jyoban Hospital of Tokiwa Foundation, Fukushima, Japan; 3 Department of Gastroenterology, Sendai Kousei Hospital, Sendai, Japan; 4 Department of Surgery, Jyoban Hospital of Tokiwa Foundation, Fukushima, Japan; 5 Department of Radiation Health Management, Fukushima Medical University, Fukushima, Japan

**Keywords:** Cancer, Public Health, Refugees, Long-Term Care, Disasters

## Abstract

The ongoing Russo-Ukrainian War and the Great East Japan Earthquake and the subsequent accident at the Fukushima Daiichi Nuclear Power Plant in Japan have many similarities, such as mass evacuation, family separation, difficulty in accessing necessary medical care, and reduced health priorities. Although several studies have reported concerns about the short-term health impacts of the war on patients with cancer, little has been noted about the long-term effects it may cause. Given the experience of the Fukushima accident, it is important to establish a long-term support system for patients with cancer in Ukraine.

More than 5 months after the Russian invasion of Ukraine, various concerns have been reported regarding its immediate impact on cancer care of patients in Ukraine. With the end of the war still uncertain, raising awareness regarding its long-term impact on patients with cancer and their treatment is important. For example, even after patients return to “normalcy,” cancer-related help-seeking behavior may change over the long term, possibly delaying consultation of symptomatic patients with cancer and declining cancer screening coverage. The reason for this is the similarity between the Great East Japan Earthquake and the Fukushima Daiichi Nuclear Power Plant accident (triple disaster) in Japan and the ongoing Russo-Ukrainian War.

The primary similarity between the two crises is mass evacuation. The ongoing war resulted in more than 4 million people evacuating Ukraine. While it is reported that some evacuees have returned after the key battlefield shifted to the east, many evacuations are likely to be prolonged, and it would be more difficult for both who left and stayed to receive support from their families or communities. In particular, families are normally the last remaining mutual aid system in an evacuation. However, because adult males were prohibited from leaving Ukraine by the National Mobilization Law, evacuations are mainly for women and children, forcing many to be separated from their families.

Similarly, after the triple disaster, many people were forced to evacuate. In particular, due to concerns about radiation, women and children were often evacuated primarily from the affected areas, and delays in or avoidance of help-seeking were observed in both those who left and those who remained ^(1), (2)^. Moreover, poor access to medical care for displaced people has been observed in various disasters, including the triple disaster ^(1)^, and the past and current conflicts seem to be no exception. What is noteworthy is that long-term health damage regarding cancer was observed after the triple disaster. In our previous studies, help-seeking was found to decline over 5 years ^(3)^, and screening coverage declined over 3 years before returning to the predisaster level ^(4)^, Despite the limited impact on infrastructure after the triple disaster, the impact on cancer care is likely to be more prolonged this time.

Considering the insights from the triple disaster, long-term support should be provided for the severely affected Ukrainian population. Specifically, for healthcare professionals who stay and work locally to identify health impact and provide timely feedback to the community, it is necessary to develop an integrated strategy by combining both population and high-risk approaches ^(5)^. In addition to awareness programs, it is meaningful for community healthcare professionals to individually approach citizens and refugees to understand their health concerns.

## Article Information

### Conflicts of Interest

Akihiko Ozaki received personal fees from MNES Inc. outside the submitted work.

### Sources of Funding

This work was supported by the Ministry of the Environment, Japan, as a Research Project on the Health Effects of Radiation.

### Author Contributions

Conception and design of the study; YK

Data collection and writing of the paper; YK and AO

Critical revision of the paper; HS, TS, and MT

All the authors read the final draft and approved it for submission.
